# The Position of Women at Cambridge

**Published:** 1920-12-04

**Authors:** 


*To the Editor of* The Hospital.

### "The Position of Women at Cambridge."

*To the Editor of* The Hospital.

Sir,—The interesting letter of "A Member of the Senate" in your issue of November 6 contained excellent arguments against the presence of women in this University, and for the maintenance of its "masculine independence." It ignored the fact that women are already in the University but not of it, and that there is no proposal to turn them out or to keep the University "for men only." The refusal to women of formal membership in the University will not diminish what he calls "the insidious influence of the opposite sex."

The admission of women to Colleges is, and will be, a question for each College; the proposed admission of women to membership of the University does not affect this question. A College could now admit a woman as a member, scholar, or fellow, but in the present state of public opinion there are few things less likely.

The assertion that demonstrators are more assiduous in their attentions to women than to men seems to me, from personal observation, the reverse of the truth. For many demonstrators "hate all women," and therefore avoid them; others are shy, and all know that any special attention paid to a woman student would be immediately noted.

The phantasy of M.D. Cantab of petticoat and trouser "rags" throws an interesting light on his "knowledge of feminine psychology," but does not merit serious discussion.

The education at this University is not directed to training either the men or the women for specifically male and female functions. There is no shred of argument in the letter of "At Cambridge Now" to back up his *ipse dixit* that the training here is "of necessity" not the best for women. As far as I know, no concrete argument based on the curricula has been brought by opponents of the admission of women to full membership, still less by the women themselves, save one—an objection to the length and critical nature of the Tripos examinations. But since this seems to rest, so far as the vague form in which it was stated allows us to judge, upon a gross exaggeration of the effects of menstruation upon the mental health of women, it is perhaps unnecessary to discuss it. But an unscientific argument of this kind may perhaps be expected to appeal to one who, like "At Cambridge Now," is so blind as to see no other signs of co-operation between men and women here at present except in dance clubs and on the Cam. That co-operation is not yet fully developed is in the main due to the "smug hypocrisy of the Victorian era" which "At Cambridge Now" deplores; though by his opposition to Report A he refuses to do what he might to end it. —I am, Sir, yours faithfully, Another Cambridge M.D.

Cambridge, November 23, 1920.

